# A Schwannoma-Hemangioma Composite Tumor as a Very Uncommon Cause of a Chest Wall Tumor in a Teenage Patient: A Case Report

**DOI:** 10.7759/cureus.81884

**Published:** 2025-04-08

**Authors:** Ewa A Bieganska, Jakub Noskiewicz, Zaneta Slowik-Moczydlowska

**Affiliations:** 1 Department of Pediatric Surgery, Medical University of Warsaw, Warsaw, POL

**Keywords:** cavernous hemangioma surgery, chest wall tumor, nerve sheath tumor, oncology pediatrics, pediatric surgery

## Abstract

Schwannoma-hemangioma composite tumors are extremely rare, with most cases described in the head and neck of adult patients. Pediatric cases involving the chest wall have not been widely reported and, therefore, present diagnostic and therapeutic challenges. We describe the case of a 14-year-old male patient who presented with a painless nodular lesion on the chest wall. After imaging studies and laboratory analysis, the consulting oncologist recommended the surgical removal of the tumor due to the indeterminate nature of the lesion. Histopathologic analysis of the excised tissue confirmed a composite schwannoma-hemangioma tumor. The patient recovered uneventfully postoperatively with no recurrence or complications at the three-month follow-up. This case highlights the importance of considering rare composite tumors in the differential diagnosis of pediatric chest wall lesions. Accurate histopathologic evaluation is essential for optimal management. Further case reports are needed to improve the understanding of the etiology and clinical behavior of such tumors.

## Introduction

Peripheral nerve sheath tumors and hemangiomas are common benign soft tissue tumors. Sporadic schwannomas account for approximately 89% of all peripheral nerve sheath tumors. They are most commonly found in the upper extremities, head and neck, trunk, and flexor surfaces of the lower extremities. These tumors are typically diagnosed in people between the ages of 20 and 50, with no gender or ethnic predilection [1,2].

Hemangiomas are the most commonly diagnosed benign soft tissue tumors in children. They occur predominantly in the head and neck region [3].

Despite the high prevalence of both tumor types, their simultaneous occurrence either as a composite tumor (known as conjoined association) or as separate tumors in close proximity but different locations (also known as discrete association) is extremely rare. According to the literature, only about 40 cases of such occurrences have been documented, mainly in the head and neck region [4-6]. Because of their rarity, the diagnosis and management of these tumors are clinical challenges.

Another critical clinical consideration is the occurrence of chest wall tumors in pediatric patients. They comprise a heterogeneous group of lesions, ranging from benign to malignant, and may arise from osseous, cartilaginous, or soft tissue structures. Common benign tumors include lipomas, fibromas, and chondromas, while malignant lesions may consist of peripheral primitive neuroectodermal tumors (PNETs), osteosarcomas, rhabdomyosarcomas, and metastases. Imaging plays a critical role in the initial assessment, with CT and MRI providing valuable information on lesion composition, extent, and potential invasiveness. Ultrasound is also a very useful diagnostic test, characterized by good resolution in pediatric patients and increased radiological safety, which is important for this group. Despite advances in imaging, definitive diagnosis often requires histopathological confirmation due to overlapping radiological features. Although chest wall tumors as a group are rare, a significant proportion are malignant and require increased oncological vigilance [7-9].

The purpose of this report is to present the case of a 14-year-old patient who was admitted to the hospital with a small nodular lesion in the chest wall region. Following surgical excision, histopathological analysis revealed features consistent with a schwannoma-hemangioma composite tumor. Given the rarity of this tumor type, particularly in the pediatric population, publishing this case aims to improve understanding of the diagnosis and management of such tumors. Such knowledge may be of benefit to pediatric surgeons and pathologists in their clinical practice.

## Case presentation

A 14-year-old previously healthy, male patient presented to the pediatric surgery clinic with the chief complaint of painless swelling in the chest region. The patient had been initially evaluated during a diagnostic workup at the oncology clinic after a nodular lesion was noted between the 10th and 11th ribs on the left side. The lesion was asymptomatic and was discovered incidentally during an outpatient evaluation for a nodule in the breast region, where glandular tissue was identified.

Physical examination revealed a palpable, mobile nodule in the chest wall measuring approximately 2×3 cm, located along the left midaxillary line between the 10th and 11th intercostal spaces, and palpable axillary lymph nodes on the right side. No other abnormalities were noted.

During the first diagnostic stay in oncology, a differential diagnosis was made to determine whether the lesion was benign, such as a lipoma or hemangioma, or whether it could be a malignant tumor such as PNETs, sarcomas, or very rare but possible germ cell tumors. To this end, imaging studies were ordered, chest X-ray, abdominal ultrasound, chest wall ultrasound, and laboratory tests, including the available tumor markers that could indicate the aforementioned PNET or germ cell tumors. The results of the markers are shown in Table 1.

**Table 1:** The results of the tumor markers AFP: alpha-fetoprotein; β-hCG: beta-human chorionic gonadotropin; LDH: lactate dehydrogenase; NSE: neuron-specific enolase

Marker	Results	Reference values	Units of measurement
AFP	1.5	<8	ng/ml
β-hCG	<0.10	<2.0 (for males)	mIU/ml
LDH	177	120-300	U/l
NSE	11.9	9-12.4	ng/ml

**Figure 1:**
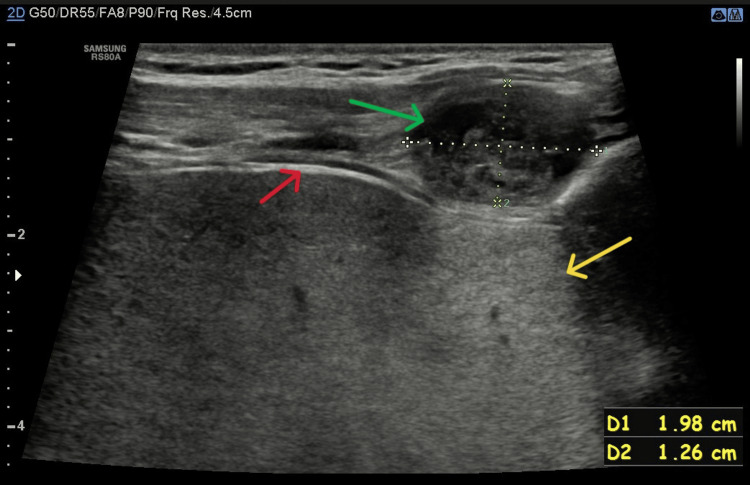
Image from ultrasound examination: lesion with cystic elements (green arrow), visceral pleura (red arrow), and posterior acoustic enhancement (yellow arrow) is presented

**Figure 2:**
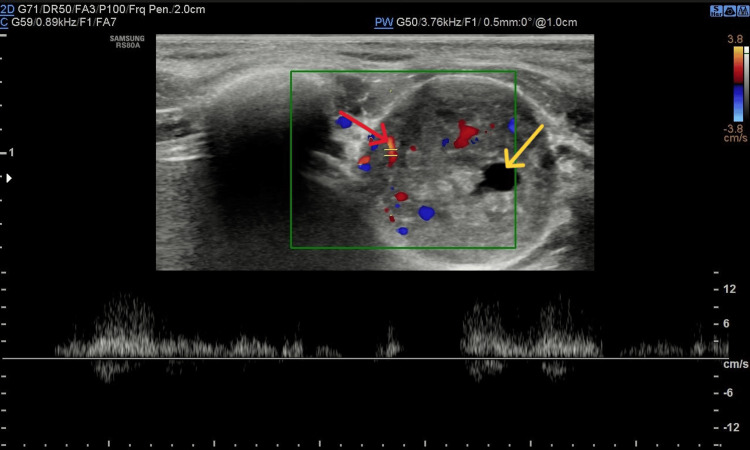
Image from Doppler ultrasonography: lesion with arterial flow (red arrow) and cystic elements (yellow arrow) is presented

**Figure 3:**
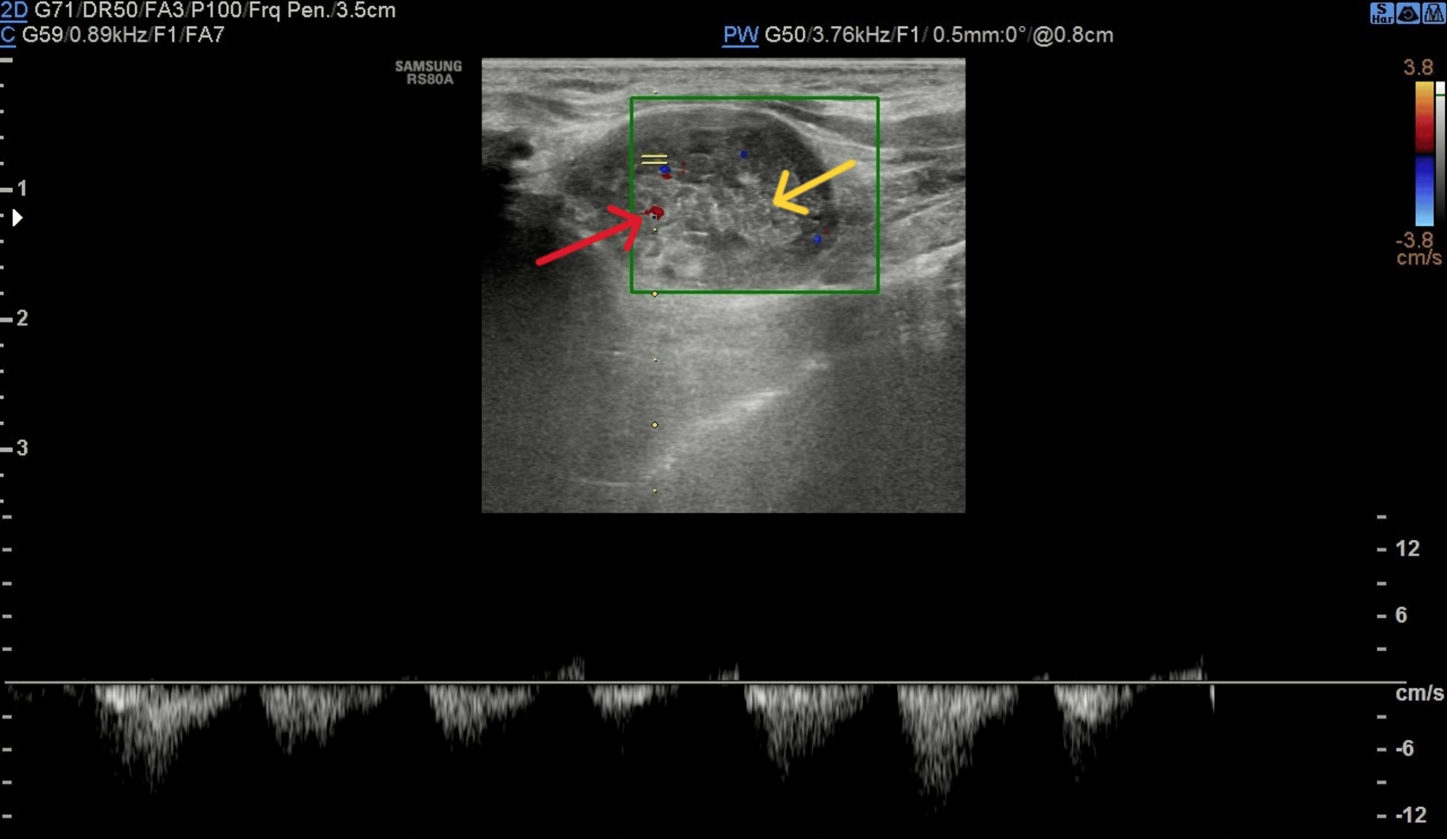
Image from Doppler ultrasonography: apart from arterial flow (red arrow) in the lesion, solid elements with heterogeneous echostructure (yellow arrow) are presented

Chest X-ray and abdominal ultrasound were unremarkable. Ultrasound of the chest wall lesion showed a focal mass measuring 20×13 mm. The lesion was described as containing both solid and cystic elements, with heterogeneous echotexture, posterior acoustic enhancement, and visible arterial flow within the lesion (pictures from ultrasound examination: Figures 1-3). Previously palpated axillary lymph nodes were normal on ultrasound.

Since the ultrasound image did not allow a clear differentiation of the lesion, as it did not show features characteristic of benign tumors such as lipoma or hemangioma, and the nodule did not show radiological features of malignancy, and tumor markers were negative, it was decided to remove the lesion in its entirety for pathomorphological examination of the lesion.

The lesion was excised under general anesthesia. Intraoperatively, a well-demarcated, encapsulated mass was observed with no connection to adjacent bone, muscle, or pleura. The lesion, which was more cohesive than the surrounding tissues and cream in color, was completely excised together with its capsule. The procedure was uneventful, with typical closure of the surgical site. The postoperative course was uncomplicated, and the patient was discharged on the first postoperative day and referred to outpatient care.

**Figure 4:**
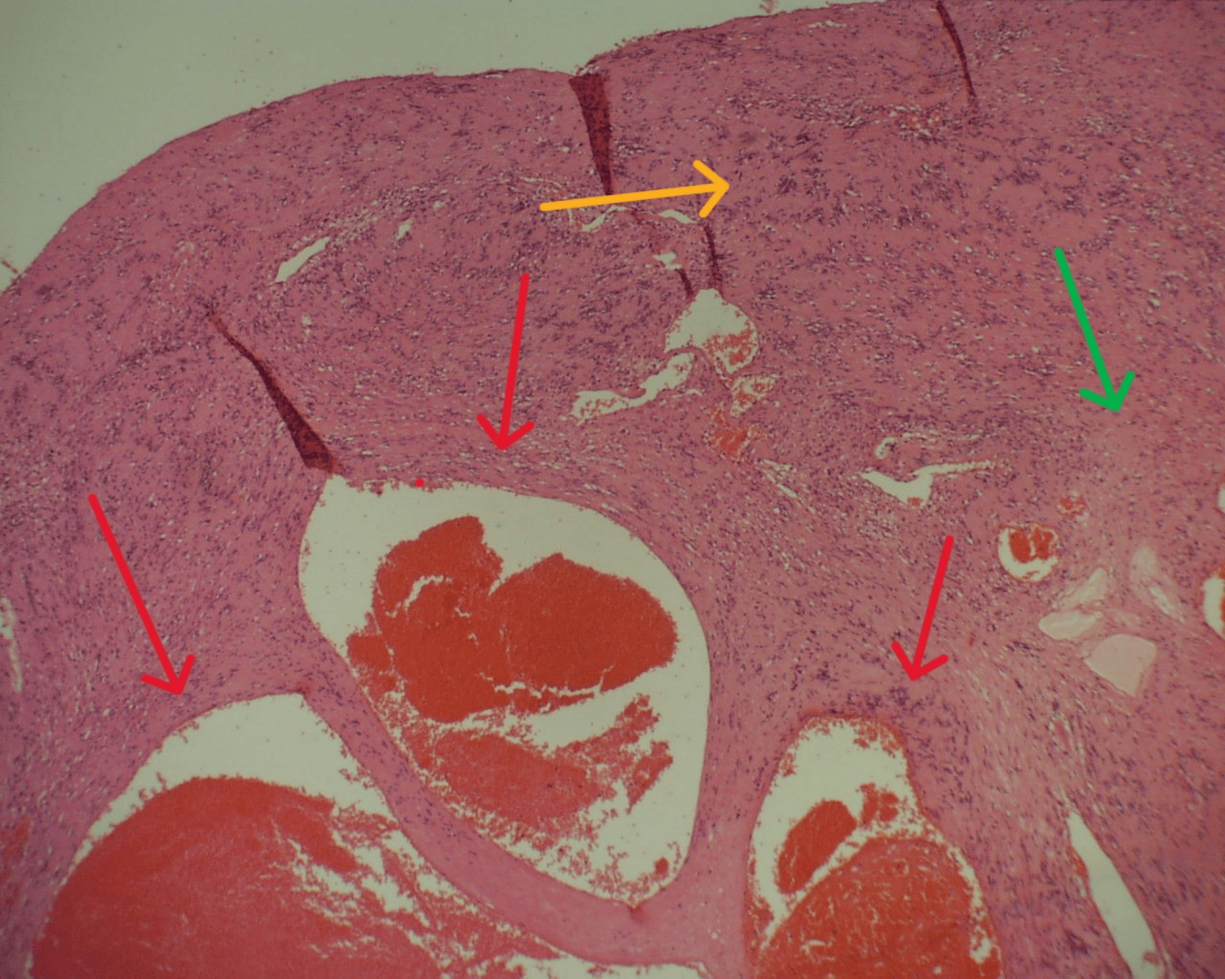
Histological preparation, HE staining, 40× magnification. Part of the lesion indicated by red arrows is comprised of dilated congested thin-walled vessels, characteristic for cavernous hemangioma. Also, the characteristic biphasic structure can be seen: Antoni A (hypercellular, yellow arrow) and Antoni B (hypocellular, green arrow) areas

**Figure 5:**
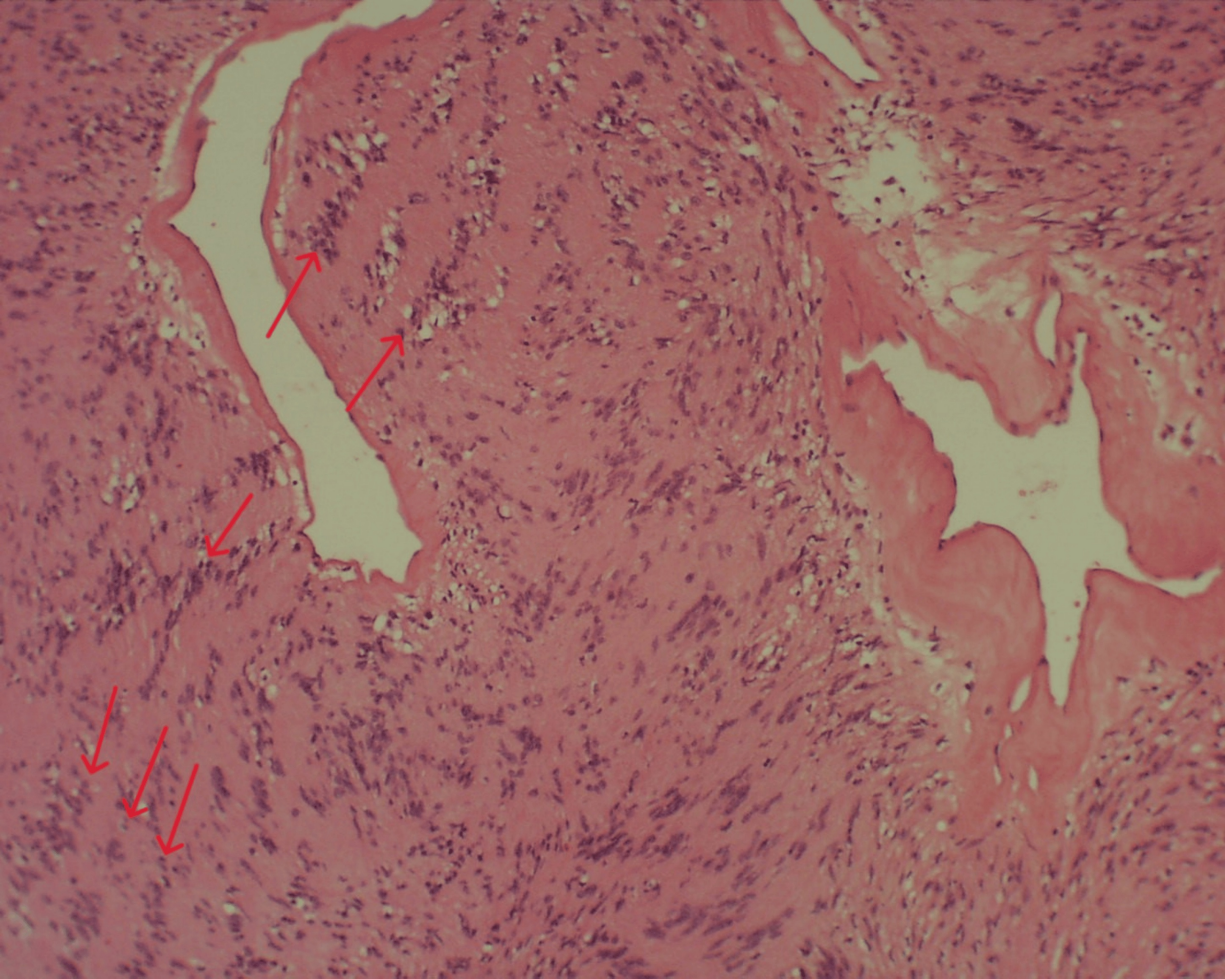
Histological preparation, HE staining, 100× magnification. Characteristic areas of nuclear palisading around fibrillary process (Verocay bodies) are indicated with red arrows

**Figure 6:**
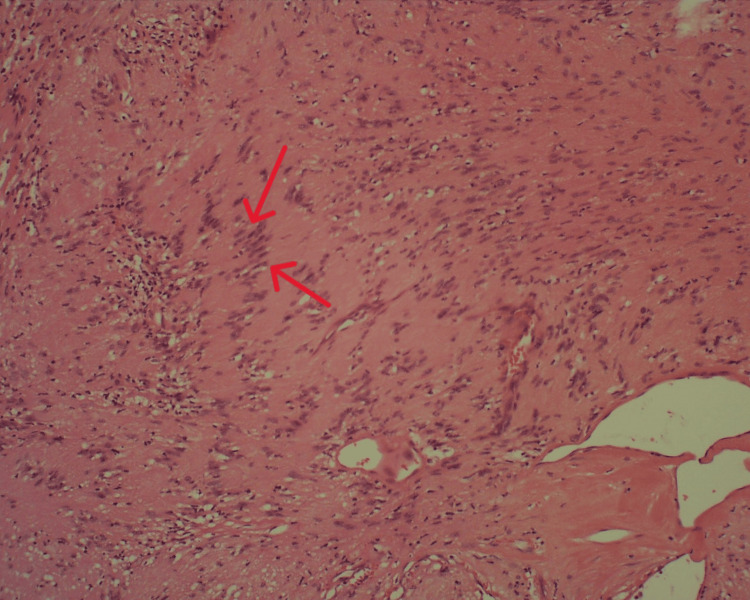
Histological preparation, HE staining, 200× magnification. Verocay bodies are indicated with red arrows

**Figure 7:**
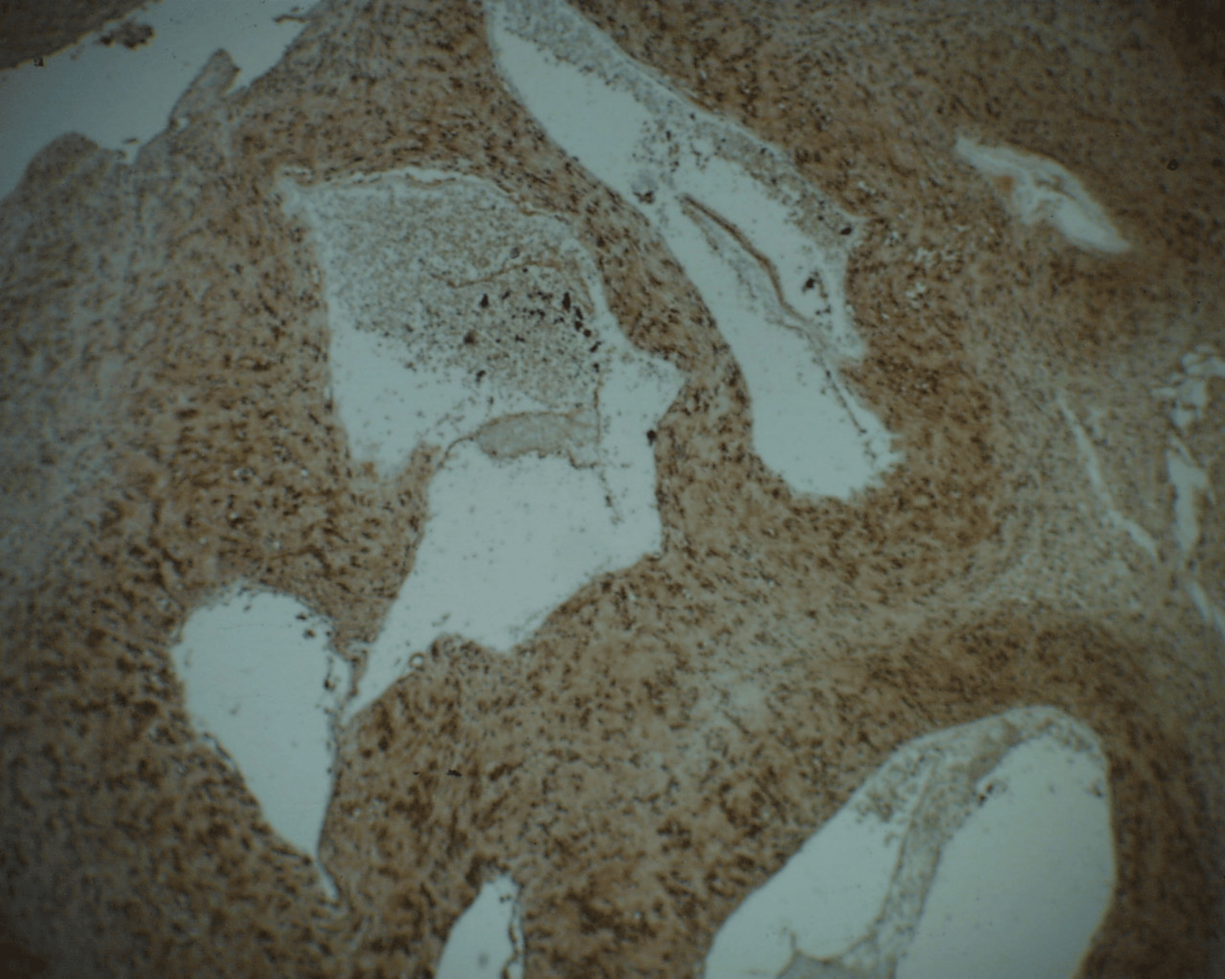
Immunostain for S100 shows nuclear and cytoplasmic staining in the cells, 200× magnification Strong and diffuse staining pattern is observed in the picture

Histopathological examination revealed features consistent with a schwannoma, characterized by the expression of S100 protein and CD56 antigen, together with dilated vascular spaces typical of a cavernous hemangioma (Figures 4-7). No other immunohistochemistry markers were performed. The tumor was removed in its entirety, and the surgical margins were negative. The overall findings support the diagnosis of a composite schwannoma-hemangioma tumor.

## Discussion

The case of a 14-year-old patient with a composite schwannoma-hemangioma tumor in the chest wall is noteworthy because of the rarity of such lesions and their atypical location. Previously reported cases of coexisting neural and vascular tumors have mainly involved adult patients, with lesions located in the head and neck region, affecting either the central nervous system or cranial nerves. In most cases, the neural tumors were schwannomas, with neurofibromas and gliomas being less commonly reported [5]. Although the coexistence of two different tumor types in a single location may be coincidental, the literature suggests that complex molecular mechanisms are more likely to underlie the simultaneous development of schwannomas and hemangiomas within a single tumor.

Proposed pathways of tumorigenesis include loss of Merlin-mediated inhibition of the MAP kinase MEK/ERK cascade, abnormalities in the PI3K/mTOR pathway, interactions between vascular endothelial growth factor (VEGF) secreted by schwannoma cells and endothelial cells, and KRIT1 gene mutations [5,6].

Identifying the molecular mechanisms responsible for the development of such tumors could be crucial in determining therapeutic strategies, particularly pharmacological approaches, in cases where complete surgical resection is not feasible due to the location of the tumor.

Another important consideration is the differential diagnosis of chest wall tumors in children, as a significant proportion of these lesions are malignant, such as Ewing's sarcoma, lymphoma, or metastatic tumors. According to review articles, benign tumors such as chondromas, hemangiomas, or the mixed tumor described in this report represent only a small percentage of all chest wall tumors in this age group [7-9]. 

Both van den Berg et al. [7] and La Quaglia [10] emphasized that although benign chest wall tumors are often incidental findings, typically identified by imaging studies, definitive diagnosis usually requires histopathological examination. Therefore, it is emphasized that in cases of pediatric chest wall tumors, biopsy or surgical resection should always be considered to establish the nature of the lesion and determine the optimal therapeutic approach.

## Conclusions

This case highlights the importance of considering rare complex tumors in the differential diagnosis of subcutaneous and chest wall lesions, even in pediatric patients. Apart from diagnostic imaging like CT, MRI, and ultrasound, accurate histopathological and immunohistochemical evaluation is essential for correct diagnosis and, thus, therapeutic management. The publication of this case is a valuable addition to the literature on schwannoma-hemangioma tumors and may be helpful in the diagnosis and management of similar cases in the future.

Further studies and case reports are needed to gain a better understanding of the etiology, clinical course, and optimal management strategies for complex tumors of this type.
